# Supplementary material for: MetaQTL: a package of new computational methods for the meta-analysis of QTL mapping experiments
Source: BMC Bioinformatics. 2007 Feb 8;8:49. doi: 10.1186/1471-2105-8-49 (PMC1808479; doi:10.1186/1471-2105-8-49)
Supplement: Additional File 7 — MetaQTL Package : jar file and tutorial. This Zip archive contains both the MetaQTL JAR file and the files of the tutorial. [file 1471-2105-8-49-S7.zip › org.inra.metaqtl/doc/org/metaqtl/class-use/Qtl.QTLCross.html]

Uses of Class org.metaqtl.Qtl.QTLCross


|  |  |  |
| --- | --- | --- |
| **Overview** \| **Package** \| **Class** \| **Use** \| **Tree** \| **Deprecated** \| **Index** \| **Help** | |  |
| PREV   NEXT | **FRAMES**    **NO FRAMES**     **All Classes** |  |


---


## **Uses of Class org.metaqtl.Qtl.QTLCross**

| Packages that use Qtl.QTLCross | |
| --- | --- |
| **org.metaqtl** |  |

Uses of Qtl.QTLCross in org.metaqtl

| Fields in org.metaqtl declared as Qtl.QTLCross | |
| --- | --- |
| `Qtl.QTLCross` | `Qtl.cross`             The QTL cross design. |

| Methods in org.metaqtl that return Qtl.QTLCross | |
| --- | --- |
| `Qtl.QTLCross` | `Qtl.QTLCross.getCopy()` |

| Methods in org.metaqtl with parameters of type Qtl.QTLCross | |
| --- | --- |
| `static double` | `Qtl.getQTLSD(double rsquare, Qtl.QTLCross cross, int mappingUnit)`             Depending on the r-square parameter and the cross design this method returns the standard deviation of the QTL position for the given mapping context. |

---


|  |  |  |
| --- | --- | --- |
| **Overview** \| **Package** \| **Class** \| **Use** \| **Tree** \| **Deprecated** \| **Index** \| **Help** | |  |
| PREV   NEXT | **FRAMES**    **NO FRAMES**     **All Classes** |  |


---
